# Soil properties, bacterial and fungal community compositions and the key factors after 5-year continuous monocropping of three minor crops

**DOI:** 10.1371/journal.pone.0237164

**Published:** 2020-08-24

**Authors:** Pu Yang, Yan Luo, Yang Gao, Xiaoli Gao, Jinfeng Gao, Pengke Wang, Baili Feng

**Affiliations:** 1 College of Agronomy, State Key Laboratory of Crop Stress Biology in Arid Areas, Northwest A&F University, Shaanxi, China; 2 Shaanxi Research Station of Crop Gene Resources & Germplasm Enhancement. Ministry of Agriculture, Shaanxi, China; 3 Agriculture Bureau of Gaochun District, Nanjing, China; ICAR-National Rice Research Institute, INDIA

## Abstract

Minor grain crops are widely cultivated in northwest China and played important roles in local economic. Soil microbes play a central role in ecological function and biological stability and related to soil quality. In order to uncover the soil microbial composition differences and the factors under 5-year continuous monocropping of three minor crops (Proso millet, Common bean and Common buckwheat) in Guan-Zhong Plain, six soil nutrimental parameters, soil pH, soil moisture content, and four soil enzyme activities were analyzed and soil microbial composition were sequenced. The results showed that after 5-years of continuous monocropping, different cover crops influenced most of soil physicochemical properties, expect soil moisture content (*P* < 0.05), the available nutrients were significant higher in proso millet soil, and the pH was significantly higher in common buckwheat soil. soil ALP, catalase and urease activities were significantly different between soils (*P<* 0.01), in which soil catalase activities were significantly lower and soil ALP and urease activities were significantly higher than that of proso millet and common buckwheat. A total of 171439 sequences, 9468 OTUs and 29 phylum for bacteria, 128920 sequences, 544 OTUs and 27 phylum for fungi were obtained. In addition, no significantly difference obtained in diversity and richness between soils (*P* < 0.05). According to relative abundance, *Proteobacteria*, *Chloroflexi*, *Gemmatimonadetes* and *Acidobacteria* were the dominant bacterial phylum in all samples, moreover, the relative abundance of *Caldiserica* was significantly different between soils (*P <* 0.05). *Ascomycota* (79.04%-90.21%) was dominant phylum in fungal community and phylum *Phragmoplastophyta* (*P <* 0.01) and *Glomeromycota* (*P <* 0.05) were significantly different between soils. Redundancy analysis indicated that available nutrients Nitrogen and Potassium are the strongest predictors in both bacterial and fungal community. In conclusion, different cover crops influenced soil nutrient properties, soil pH and soil microbial composition, and continuous monocropping decreased soil fertility condition. Moreover, Common bean and Common buckwheat were more sensitive to monocropping treatment.

## Introduction

Proso millet (*Panicum miliaceum* L.), common bean (*Phaseolus vulgaris*) and common buckwheat (*Fagopyrum esculentum* Moench) are three important minor grain crops that are beneficial to human health [1]. They are widely cultivated in northwest China and playing important roles in the development of local agricultural economy [2]. There are multiple cropping systems in the production of proso millet, common bean and common buckwheat, including crop rotation and continuous monocropping. According to previous studies, continuous monocropping has a significant negative impact on crop yield and soil quality [3, 4], and it was related to soil capacity to suppress the soil borne pathogen in soybean [5], decreased in sensitive soil quality parameters in cotton [6] and yet had been reported to reduce microbial diversity significantly, decreasing the fertility of soil in wheat [7] and lower the genetic diversity level and simplex community structure in peanut [8]. However, by the end of 2013, China had 130 Mha of arable land, of which about 124 Mha was planted continuously [9], and provided great potential to increase production.

Soil is the habitat of a diverse and heterogeneous range of microorganisms [10]. Microbes are particularly important in agricultural soils, due to a central role in ecological function and biological stability [11, 12], and related to soil quality [13, 14]. The soil microbial composition respond to agricultural managements [12] and land use [15] and so on, in the meantime, any change they experience are likely reflecting in the functional integrity of soil [16]. The bacterial communities differed between rotation and continuous cropping of corn [17], wheat [16], and peanut [8] and so on. The bacterial community composition was influenced by plant production [7], soil pH [18, 19], soil water, soil organic matter and soil C/N [20] and soil enzyme activities [21] and the community composition of fungi, especially the arbuscular mycorrhiza fungi, was influenced by rotation of plants [22] and soil parameters more than site [23]. And, previous studies reported the main factors of soil microbial composition are plot site [24], land management [16], farming system [7, 17], soil physicochemical parameters [25, 26], and cover crops [8].

Moreover, soil microbial composition and the main factors under continuous monocropping of proso millet, common buckwheat and common bean in northwest China are still unclear. And it is necessary to study the diversity and composition differences of soil microorganisms and the key factors after cropping different type of cover crops. Therefore, the objective of this study was to: (1) uncover the structure, richness and diversity of soil bacterial and fungal community under a 5-year continuous cropping of proso millet (MZ), common bean (YD) and common buckwheat (QM), respectively. (2) compare the soil nutrition content and enzyme activity differences among three soils. (3) find out the relationship between soil parameters and soil microbial community, and find the key factors of soil microbial composition in our experiment.

## Materials and methods

### Site description

The experiment site is a trial plot in north campus of the Northwest A & F University, Yangling, Shaanxi, China (108°05´E, 34°17´N), which belongs to semi-humid drought-prone areas and loam soils which belongs to semi-luvisols soil. The average annual temperature is 12.9 °C. The mean annual precipitation is 551.0 mm of which over 60% received during July to September, while the average annual evaporation is 1400 mm.

At the very beginning of the experiment in 2010, soil properties were investigated. The 0–20 cm layer soil contained 1.59 × 10^6^ kg·ha^-1^s and (1–0.05 mm), 2.067×10^7^ kg·ha^-1^ silt (0.05 ~ 0.001 mm) and 4.24×10^6^ kg·ha^-1^ clay (<0.001 mm), with a pH (H_2_O, 1: 2.5) of 8.3. Moreover, soil contained total nitrogen (TN) 3.286×10^4^ kg·ha^-1^, total phosphorus (TP) 2.7825×10^4^ kg·ha^-1^, total potassium (TK) 5.353×10^5^ kg·ha^-1^, available nitrogen (AN) 2308.15 kg·ha^-1^, available phosphorus (AP) 869.2 kg·ha^-1^, available potassium (AK) 4208.2 kg·ha^-1^, and soil organic carbon 322.8 kg·ha^-1^.

### Soil sampling

The experiment carried out from 2010 to 2014 and 3 different species of monocropping as follows: The proso millet (MZ) variety Neimei-5, common bean (YD) variety Y0503 and common buckwheat (QM) variety Xinong 9920 were monocropped for 5 years respectively. The plants were planted in a 10 square meters cell (2m×5m), in a randomized complete block design, with four replicates. With no fertilization providing and a conventional field management during growing seasons.

Soil in the plot is randomly mixed at 3 points (0–20 cm), and the "quadruple method" and then forms 1.5 kg of soil samples. After the crops was harvested in 2014, the samples were taken for determining the physical and chemical properties of the soil. The crop rhizosphere soils samples for measuring soil enzyme activities were collected in 15/7/2014. All soil samples were decontaminated, naturally air-dried, and divided into two parts, partly ground through 0.8 mm sieves for the testing of soil properties and another part ground through 0.16 mm sieves for soil enzyme activities. The samples for soil community determination were collected in 15/7/2014 ground through 0.16 mm sieves and then stored at -80 °C refrigerator for testing.

### Soil nutrient contents and soil moisture content analysis

TN was determined by Kjeldahl digestion—continuous flow analyzer; TP was determined by H_2_SO_4_ digestion—molybdenum sulfate anti-colorimetric method; TK was determined by sodium hydroxide melting (450 °C)—flame photometry. The AN was determined by 1.0 mol·L^-1^ KCl extraction-AA3 continuous flow analyzer. The AP was determined by 0.5 mol·L^-1^ NaHCO_3_ extraction—molybdenum sulfate anti-colorimetric method; and the AK was extracted with 1.0 mol·L^-1^ NH_4_OAc—Determined by flame photometry; pH is determined by leaching-potential method (soil to water ratio is 1:2.5) [21]. Soil moisture content (SMC) was calculated as below:

$$SMC=\frac{W_{f}-W_{d}}{W_{d}-W_{t}}\times 100\%$$ (1)

Where the *W*_*f*_ for fresh soil weight, *W*_*d*_ for drought soil weight, *W*_*t*_ for aluminum box weight.

The soil enzyme activity is measured as follows: phenyl phosphonate colorimetric method for alkaline phosphatase activity (ALP); catalase UV spectrophotometry for catalase activity; urease colorimetric method for urease activity; 3,5-dinitrosalicylic acid colorimetric method for sucrose activity [26].

### The macro genome sequencing of soil bacteria and fungi

Total genomic DNA from soil samples were extracted with TIANamp Soil DNA Kit (TIANGEN BIOTECH Inc., Beijing, China) following the manufacturer’s instructions. DNA concentration and purity was monitored on 1% agarose gels. The universal prokaryotic primers 338F (5´-ACTCCTACGGGAGGCAGC A-3´) and 806R (5´-GGACTACHVGGGTWTCTAAT-3´) were used to amplify the V3 and V4 regions of bacterial 16S rRNA gene. ITS5 (5′-GGAAGTAAAAGTCGTAACAAGG-3′) and ITS2 (5′-GCTGCGTTCTTCATCGATGC-3’) of fungi-specific internal transcribed spacer (ITS1) region [27]. These primer pairs were all barcoded. For 16S rRNA gene and ITS region PCR amplification, the PCR reactions were carried out with the ABI GeneAmp^®^ 9700 (ABI, Carlsbad, CA). The PCR mixture system contained 4 μL of 5 × FastPfu Buffer, 2.5 mM dNTPs, 5 μM of each primer, 0.4 μL of Taq polymerase, approximately 10 ng of DNA template, add ddH_2_O to a final volume of 20 μL. For the 16S V3+V4 rRNA gene, the PCR steps were: an initial denaturation temperature of 95 °C for 2 min, followed by 28 cycles of denaturation at 95°C for 30 s, annealing at 59.3°C for 30 s, polymerization at 72°C for 45 s, and a final elongation at 72°C for 10 min. For ITS gene, the steps were an initial denaturation at 95°C for 2 min, 34 cycles of denaturation at 95°C for 30 s, annealing starting at 59.3°C for 30 s, elongation at 72°C for 45 s, and a final extension at 72°C for 10 min. Then the PCR products were detected by 1% agarose gel electrophoresis, recovered by gel-recovery using AxyPrepDNA gel recovery kit (AXYGEN, Silicon Valley, CA), and eluted by Tris-HCl and detected by 2% agarose gel electrophoresis again [21].

According to the initial quantitation of the electrophoresis, quantitative PCR products quantified using the QuantiFluor^™^-ST Blue Fluorescence Quantitation System (Promega, Madison, WI, USA). The products mixed in the appropriate proportions according to the sequencing requirements of each sample. Miseq libraries were constructed and sequenced. Sequencing was performed on a genome analyzer Illumina Miseq PE300 (Illumina, Inc., CA, USA) with two paired-end read cycles of 101 bases each.

### Data analysis

The sequencing data filtered by quality and analyzed using Trimmomatic and FLASH software. The sequences were clustered into Operational Taxonomic Units (OTUs) using Usearch (version 7.1 http://drive5.com/uparse/) with 97% similarity. The final tables and histograms with basic OTUs were generated using biom-format package. The Mothur software [28] (version v.1.30.1 http://www.mothur.org/wiki/Schloss_SOP#Alpha_diversity) was used to analysis α-diversity indexes (Chao, Ace, Simpson, Shannon and coverage) by 97% similarity of OTUs. The R packages were used to analysis Meta-sequencing data and figures. The taxonomic classifications of bacteria and fungi were performed based on the Silva database [29] (Release119, http://www.arb-silva.de) and Unite database [30] (Release 6.0, http://unite.ut.ee/index.php), respectively. SPSS 16.0 for soil physicochemical and enzyme activity data analysis (n = 3), Canoco 5 for Redundancy analysis (RDA) and Origin 9.5 for figures.

## Results

### Soil physicochemical characters of different soils

Soil total nitrogen (TN), total phosphorus (TP), total potassium (TK), available nitrogen (AN), available phosphorus (AP), available potassium (AK), pH and soil moisture content (SMC) were investigated and analyzed after 5-years of monocropping of different cover crops (Table 1). All eight indexes were significantly different between samples; except SMC (*P* < 0.05). Soil TN (2.313–2.568 ×10^4^ kg ha^-1^), AN (497.1–522.3 kg ha^-1^), AP (132.5–197.4 kg ha^-1^), and, AK (2625–3321 kg ha^-1^) of MZ were significant higher than that of QM and YD (*P* < 0.05). The TP (1.041–1.075×10^4^ kg ha^-1^) of QM was significant higher than that of YD, however, the TK (1.008–1.043 ×10^6^ kg ha^-1^) of YD was significant higher than that of QM (*P* < 0.05). The pH (8.73–8.82) of QM was significantly higher than MZ and YD.

**Table 1:** Main soil chemical properties in three soils.

Soil ^a^	MZ	YD	QM	LSD_0.05_ ^b^
**TN** ^c^ **(kg ha**^**-1**^**)**	2.568×10^4^ ± 238.5 ^d^ a ^e^	2.303 ×10^4^ ± 1.009 ×10^4^ b	2.311 ×10^4^ ± 1272 b	S
**TP (kg ha**^**-1**^**)**	1.041 ×10^4^ ± 874.5 ab	1.012 ×10^4^ ± 477 b	1.075×10^4^ ± 238.5 a	S
**TK (kg ha**^**-1**^**)**	1.043 ×10^6^ ± 1590 a	1.044 ×10^6^ ± 4770 a	1.008 ×10^6^ ± 3.339 ×10^4^ b	S
**AN (kg ha**^**-1**^**)**	522.3 ± 8.745 a	494.8 ± 8.745 b	497.1 ± 10.34 b	S
**AP (kg ha**^**-1**^**)**	197.4 ± 27.83 a	143.1 ± 33.39 b	132.5 ± 0.00 b	S
**AK (kg ha**^**-1**^**)**	3321 ± 104.5 a	2626 ± 89.83 b	2625 ± 10.34 b	S
**pH**	8.73±0.42 b	8.74±0.42 b	8.82±0.21 a	S
**SMC (%)**	11.43±1.74 a	11.16±0.09 a	11.01±1.18 a	NS

^a^MZ = continuous cropping proso millet for 5 years, YD = continuous cropping common bean for 5 years, QM = continuous cropping common buckwheat for 5 years.

^b^LSD_0.05_ = least significant difference at *P* = 0.05, NS = non-significant, S = significant.

^c^TN = total nitrogen, TP = total phosphorus, TK = total potassium, AN = available nitrogen, AK = available potassium, AP = available phosphorus, SMC = soil moisture content.

^d^Mean ± standard deviation (n = 3).

^e^Different letters indicate significantly different between treatments (*P* < 0.05) according to Duncan’s multi-range test.

### Four soil enzyme activities of different soils

**Fig 1:**
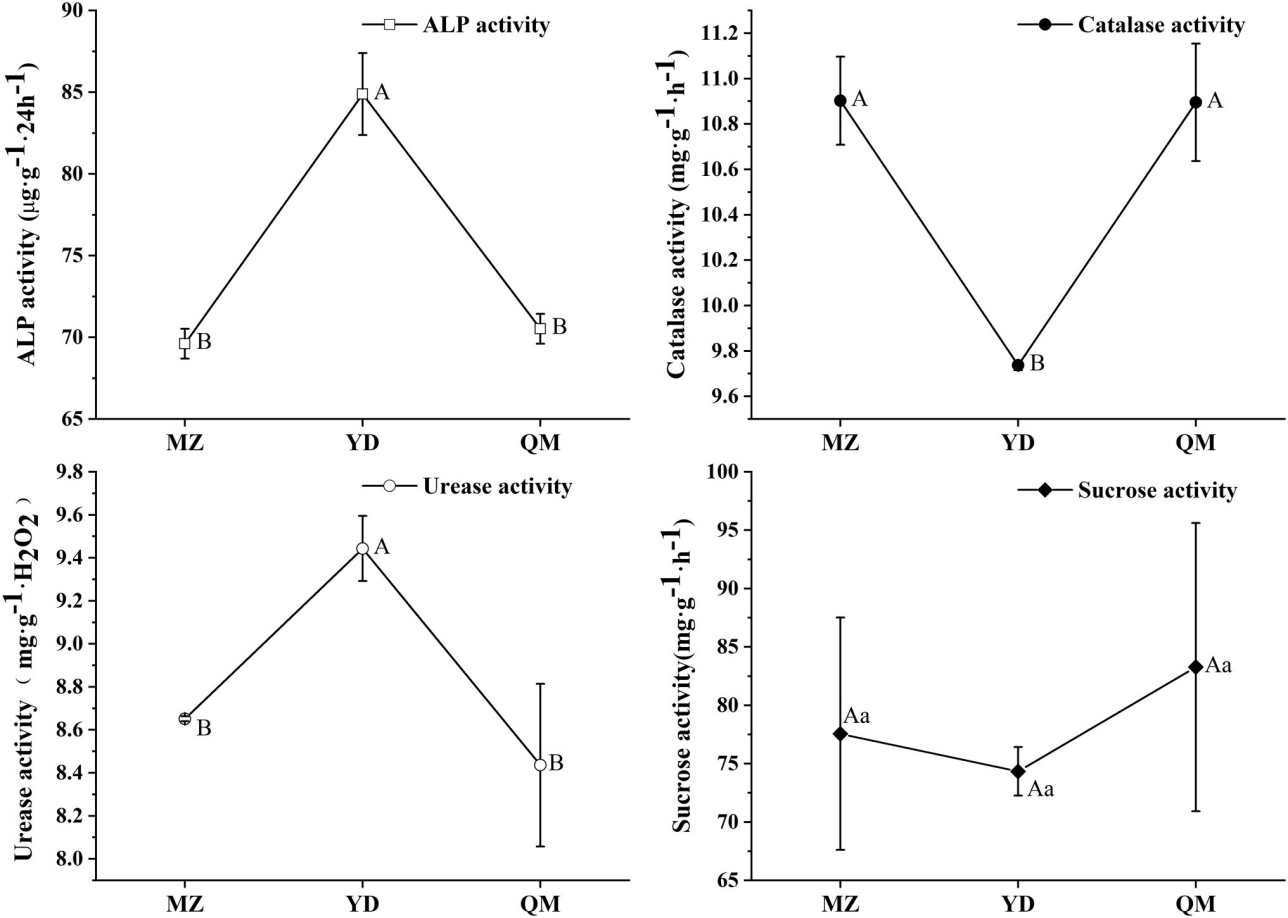
Soil alkaline phosphatase (ALP) activity (A), soil urease activity (B), soil catalase activity (C), and soil sucrose activity (D) of three soils. MZ = continuous cropping proso millet for 5 years, YD = continuous cropping common bean for 5 years, QM = continuous cropping common buckwheat for 5 years. Bars within sampling dates topped by the same letter are not significantly different according to Duncan's test at *P*<0.05.

Four soil enzyme activities were measured at 15/7/2014, and ANOVA analysis of the data was at the 0.01 level (Fig 1). Soil Alkaline phosphatase (ALP) activity (Fig 1A) in three samples was between 70–90 μg g^-1^ 24h^-1^ and the activity in YD was extremely higher than the other two soils. Soil catalase activity (Fig 1B) showed stark contrast pattern to ALP, the contents were between 9.6–11.2 mg g^-1^ H_2_O_2_, and the content in YD was significantly lower than the other two. Soil urease activity (Fig 1C) was between 8.0–9.8 mg g^-1^ h^-1^, and the activity in YD was extremely higher than the other two soils. Soil sucrose activity (Fig 1D) was no significant differences between soils, and raged from 65 to 95 mg g^-1^ h^-1^.

### Basic sequencing data of three soil

Soil samples were sequenced by metagenome sequencing technology, in each stage, samples were analyzed with three replicate. There were 171549 original sequences, 171439 active sequences generated in bacteria, 142534 original sequences, and 128920 active sequences generated in fungi. According to 97% similarity, the sequences resulted in 9468 total OTUs for bacteria and 544 total OTUs for fungi. The rarefaction curve (Amato *et al*., 2013) (S1 Fig) proved that the coverage of sequencing was reasonable, and continue sequencing will no longer produce more new OTUs. In addition, the Shannon index (Table 2) and the Shannon-Wiener curve (S2 Fig) showed that the amount of sequencing data was large enough to reflect the vast majority of microbial information in the samples.

**Table 2:** Species richness and diversity indices of soil bacterial and fungal community in different soils.

	Soil ^a^	OTU	Ace	Chao 1	Shannon	Simpson
**Bacteria**	**MZ**	1311	1225.3±44.0 ^b^ A ^c^	1240.4±54.8 A	5.8333±0.4779 A	0.0105±0.0102 A
	**YD**	1347	1210.6±107.5 A	1217.9±104.4 A	6.0333±0.2836 A	0.0073±0.0056 A
	**QM**	1273	1197.8±46.9 A	1229.0±64.6 A	6.0000±0.0520 A	0.0056±0.0003 A
**Fungi**	**MZ**	85	67.0±15.2 a	69.3±13.6 a	1.5889±0.6919 a	0.4256±0.2336 a
	**YD**	95	71.2±20.4 a	67.9±18.3 a	2.0355±0.0927 a	0.2625±0.0306 a
	**QM**	85	74.6±9.1 a	75.9±14.5 a	1.4778±0.5434 a	0.4264±0.1782 a

^a^MZ = continuous cropping proso millet for 5 years, YD = continuous cropping common bean for 5 years, QM = continuous cropping common buckwheat for 5 years.

^b^Mean ± standard deviation (n = 3).

^c^Different capital letters indicate significant (*P < 0*.*05*) differences between soils in bacterial community, whereas different small letters denote significant (*P < 0*.*05*) differences between soils in fungal community according to Duncan’s multi-range test.

**Fig 2:**
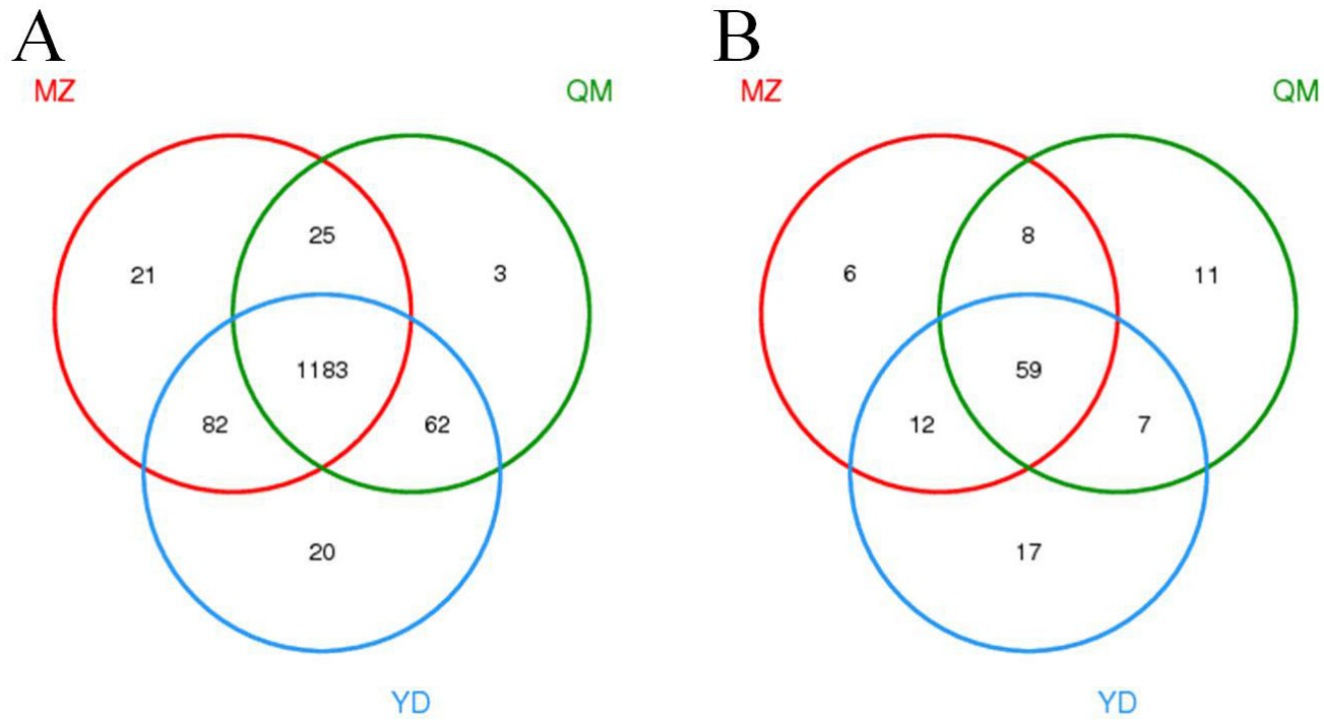
Venn diagram of bacterial (A) and fungal (B) communities in three soils. MZ = continuous cropping proso millet for 5 years, YD = continuous cropping common bean for 5 years, QM = continuous cropping common buckwheat for 5 years.

There were 1311, 1273 and 1347 total bacterial taxa from MZ, QM and YD soils, and accounting for 93.9%, 91.2% and 96.5% of the total bacterial taxa, respectively (Fig 2A). Twenty-one unique taxa (unique taxa were identified as unique that were found in all three replicates of one treatment but not in any of the triplicates of the other treatment.) in MZ soil, 3 unique taxa in QM soil, and 20 unique taxa in YD soil were detected. In all bacterial species-level taxa, 1183 (84.7%) were shared together. As for fungi, there were 85, 85 and 95 total fungal taxa from MZ, QM and YD soils, and accounting for 70.8%, 70.8% and 79.2% of the total fungal taxa, respectively (Fig 2B). Six unique taxa (7.05%) in MZ, 11 unique taxa (12.9%) in QM, and 17 unique taxa (17.9%) in YD were detected. In all fungal taxa, 59 (49.2%) were shared together.

### Soil microbial community distribution of three soil

#### The α-diversity of bacterial and fungal community in three soils

The taxa number, the richness estimators and the diversity indices of soil samples were showed in Table 2. The taxa number, Ace estimator, Chao estimator, the Shannon index, and the Simpson index had no difference between different soils (Table 2).

#### Bacterial community distribution of three soils

**Fig 3:**
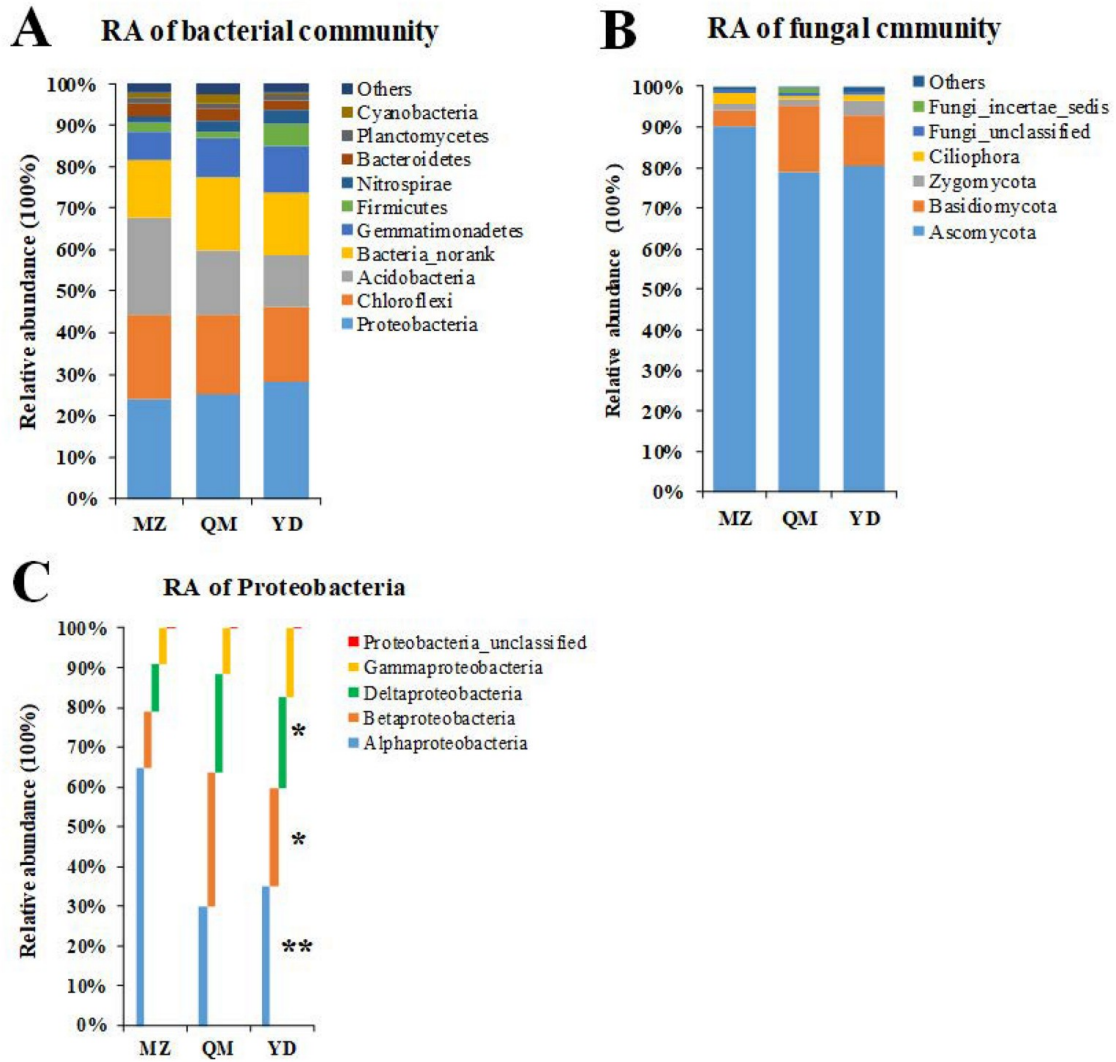
The RA distribution of soil bacterial (A) communities, fungal (B) communities and phyla *Proteobacteria* community (C) in three different soils. MZ = continuous cropping proso millet for 5 years, YD = continuous cropping common bean for 5 years, QM = continuous cropping common buckwheat for 5 years. RA = relative abundance. * = Significant difference at *P* <0.05 level, ** = Significant difference at *P* <0.01 level.

The bacterial community structure (Fig 3A) varied with different cover crops. The bacterial species obtained from MZ, QM, and YD field soils were occupied in 29 phyla by phyla level (include *bacteria-norank* and *bacteria-unclassified*). MZ soil contained all 29 phylum, QM soil (lack *Candidate_division_BRC1*) and YD soil (lack of *Chlamydiae*) contained 28 phylum. The 29 phylum were divided into two parts by relative abundance (RA). The first part is the 10 dominant phylum (RA > 1%) of the community (occupied 97.69, 97.50 and 97.90% in total) and the second part is the other 19 minor phylum (RA < 1%) of the community (occupied 2.31, 2.50 and 2.10% in total) in MZ, QM and YD soil, respectively. The top 10 phylum in MZ, QM, and YD soils were identical, although their RA and distribution were different, and the RA of different phyla was *Proteobacteria* (24.18, 25.18 and 28.00%), *Acidobacteria* (23.45, 15.29 and 12.38%), *Chloroflexi* (20.08, 19.20 and 18.33%), and *Bacteria_norank* (13.99, 17.65 and 14.85%), *Gemnatimonadetes* (6.79, 9.46 and 14.85%), *Bacteroidetes* (2.92, 2.76 and 2.06%), *Firmicutes* (2.06, 1.76 and 5.43%), *Nitrospirae* (1.68, 2.59 and 3.45%), *Planctomycetes* (1.63, 1.43 and 1.44%), and, *Cyanobacteria* (0.92, 2.19 and 0.56%), respectively. In addition, there were *α-*, *β-*, *γ-*, and *δ-Proteobacteria* and *Proteobacteria_unclassified* in phyla *Proteobacteria* and the RA of *α- Proteobacteria* (*P*<0.01), *β-Proteobacteria*, and *δ- Proteobacteria* accounted for a significant difference in *Proteobacteria* phyla level (*P*<0.05) (Fig 3C, S1 Table) between different soils.

Despite the inconsistent ranking of bacterial communities, the total taxa number of QM soil was significantly lower than that of MZ and YD. However, ANOVA analysis of RA showed no significant difference between bacterial phylum between different soils, except, *bacterial_unclassified* and *Caldiserica* (S2 Table, *P* < 0.05).

#### Fungal community distribution of three soils

The fungal community was classified into phyla level (Fig 3B). Twenty-seven phylum were obtained in fungi community, MZ obtained 23 phylum, QM obtained 23 phylum and YD obtained 26 phylum. There are quiet many phylum were the same in different soils, though there were quite a few phylum appeared uniquely. In details, phylum *Heterolobosea* and *Ochrophyta* were only obtained in YD soil, phyla *Metazoa* was only obtained in QM soil, and phylum *WIM5* and *Amoebozoa_unclassified* were did not occurred in QM soil only (S2 Table). Moreover, there are 4 dominant fungal phylum whose RA was over 1%. The ranking of these 4 fungal phylum was inconsistent in three soils. In MZ, QM and YD soils the RA of different phyla was *Ascomycota* (90.21%, 79.04%, and 80.43%), *Basidiomycota* (3.90%, 16.40%, and 12.37%), *Zygomycota* (1.72%, 1.39%, and 3.55%), and *Ciliophora* (2.39%, 0.83%, and 1.48%), respectively. The others were not classified into specific phyla, including *Fungi_unclassified* (1.03%, 0.87%, and 0.80%), *Fungi_incertae_sedis* (0.06%, 1.04%, and 0.12%), respectively. In addition, the RA of fungal phylum have no significant differences between samples, except *Phragmoplastophyta* (*P* < 0.01), *Glomeromycota* (*P* < 0.05) and *Fungi_incertae_sedis* (*P<* 0.01) (S2 Table).

### Cluster analysis of bacterial and fungal communities

**Fig 4:**
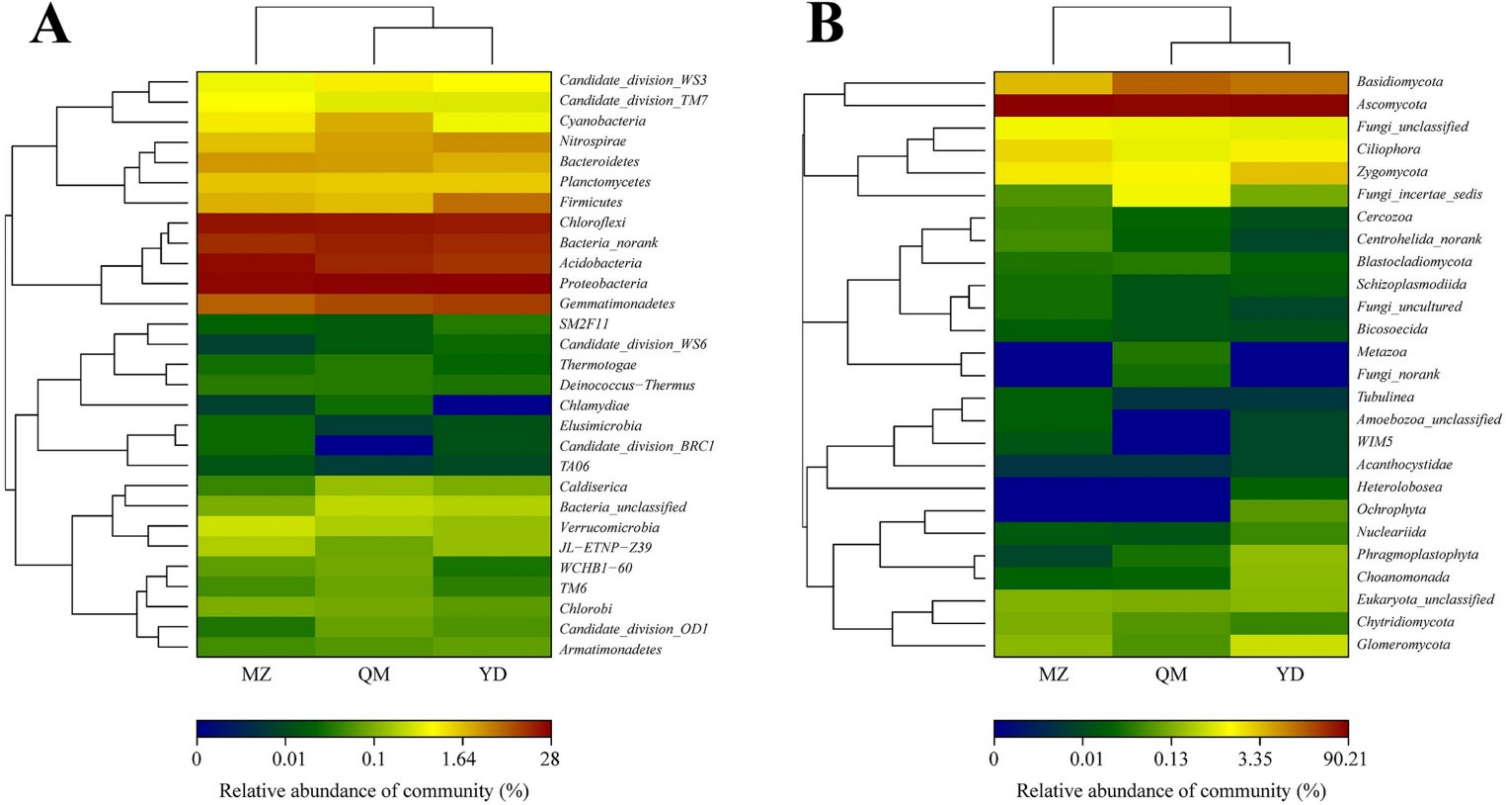
Heat map of soil bacterial (A) and fungal (B) communities of three soils. MZ = continuous cropping proso millet for 5 years, YD = continuous cropping common bean for 5 years, QM = continuous cropping common buckwheat for 5 years. Different Color indicates the relative abundance of the phyla in the total community.

As shown in Fig 4, the similarity and abundance exhibited in a heat map. The red color illustrated higher abundance, and the blue color expressed lower abundance. In addition, the heat map describes the clustering analyses of the samples, bacteria (Fig 4A) and fungi (Fig 4B), which was made at the phyla level. In Fig 4, the three soils were divided into two groups, of which the soil microbial structure (both bacteria and fungi) of QM and YD was more similar and was divided into one branch.

### Correlation analysis of abundant phylum with soil physicochemical parameters, soil enzyme activities and diversity indexes

**Fig 5:**
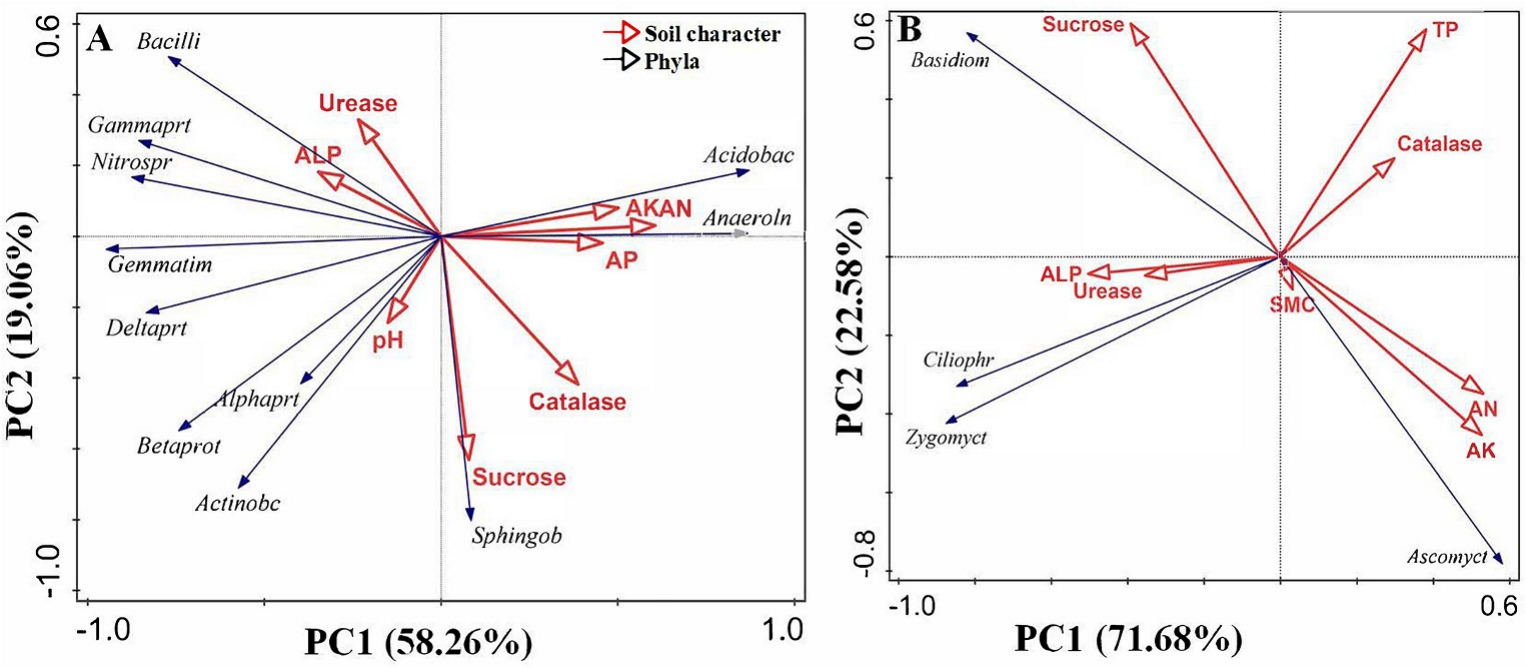
Redundancy analysis showing variation in abundant soil bacterial (A) and fungal (B) phylum explained by soil physicochemical parameters and soil enzyme activities of three soils. TN = total nitrogen, TP = total phosphorus, TK = total potassium, AN = available nitrogen, AK = available potassium, AP = available phosphorus, SMC = soil moisture content, ALP = alkaline phosphatase. Phyla with first 8 initials.

Redundancy analysis (RDA) was performed to study the effect of environmental variables (Top eight in 12 soil physicochemical parameters and soil enzyme activities) on abundant phyla (Fig 5A and 5B) (RA > 1%, and *Proteobacteria* in order level). The first two axes of RDA explain 58.26 and 19.06% for bacterial (Fig 5A) and 71.68 and 22.58% or fungal (Fig 5B) of the total variation in the data, respectively. According to Monte Carlo analysis, abundant bacterial phyla were more alike and related to AN (*p* = 0.068), and explained 25.5% variation, however, abundant fungal phyla were significantly related to AK and AN (*p* = 0.068 and *p* = 0.098), and explained 26.6 and 25.3% variation, respectively. Moreover, pH explained 6.3 and 7.5% of total variation in bacterial and fungal community (*p* = 0.5 and *p* = 0.702), respectively. In Fig 5A, abundant bacterial phyla were separated by soil characters, in which pH was significantly positively correlated with *Actinobacteria* and *α-Proteobacteria* and *β-Proteobacteria*, and AN, AK and AP were strongly correlated (positive or negative) to *Acidobacteria*, *Anaerolineae*, *γ-Proteobacteria*, *δ-Proteobacteria*, *Nitrospirae* and *Gemmatimonadetes*. As for fungi (Fig 5B), AN, AK and sucrose activity were significantly related to *Ascomycota*, otherwise, *Ciliophora* and *Zygomycota* were more related to TP, ALP, urease and catalase activities.

Spearman Correlation were assessed between abundant of bacterial and fungal phylum (RA > 1%), soil physicochemical parameters, soil activities, and soil microbial community α-diversity indexes (Table 3 and S3 and S4 Tables). Both soil bacterial or fungal phylum had no significant correlation with soil TK, AP and pH (S3 and S4 Tables). Moreover, *δ*-*Proteobacteria* was negatively correlation with TN and AN (*r* = -0.686 and -0.753, respectively, *P <* 0.05). *Gemmatimonadetes* and *Nitrospirae* were negatively correlation with AN (*r* = -0.795, *P <* 0.05 and *r* = -0.837, *P <* 0.01, respectively). As for SMC, fungal phylum had no extremely correlation with it, however, *Gemmatimonadetes* and *γ-Proteobacteria* of bacterial phylum had extremely negative correlation with SMC (*r* = -0.683 and -0.717, *P <* 0.05, respectively) (Table 3). Soil bacteria phylum had no significant correlation with soil enzyme activities (S3 Table), though fungal phyla *Ascomycota* had significant negative correlation with ALP and sucrose activities (*r* = -0.714 and -0.683, *P* <0.05, respectively), *Basidiomycota* and *Zygomycota* had positively significant correlation with ALP activity (*r* = 0.681 and 0.714, *P <* 0.05, respectively) (Table 3).

**Table 3:** Spearman’s rank correlations (r) between abundant taxa (RA >1% in all samples combined) and soil properties.

	Taxa ^a^	ALP ^b^	Sucrose	TN	TP	AN	AK	SMC
**Bacteria**	**Acidobacteria**	-0.336	0.033	0.377	0.183	0.51	0.209	0.367
	**Bacteria_norank**	0.168	0.633	0.167	0.35	0.059	-0.243	0.133
	**Bacteroidetes**	-0.176	0.5	0.251	0.267	0.151	0.126	0.633
	**Chloroflexi**	-0.034	-0.033	0.494	0.067	0.552	-0.084	-0.067
	**Firmicutes**	0.16	-0.117	-0.393	-0.533	-0.561	0.218	-0.217
	**Gemmatimonadetes**	0.412	-0.3	-0.661	-0.35	-0.795 * ^c^	-0.427	-0.683 *
	**Nitrospirae**	0.496	-0.283	-0.653	-0.367	-0.837 **	-0.586	-0.517
	**Planctomycetes**	0.126	-0.017	0.218	0.2	0.368	-0.251	-0.117
	**Proteobacteria**	0.143	-0.083	-0.293	-0.167	-0.418	0.025	-0.183
	**α-Proteobacteria**	0.034	0.1	0.243	-0.433	0	0.36	0.083
	**β-Proteobacteria**	0.218	-0.067	-0.561	0.133	-0.636	-0.435	0
	**δ-Proteobacteria**	0.412	-0.25	-0.686 *	-0.1	-0.753 *	-0.519	-0.617
	**γ-Proteobacteria**	0.252	-0.367	-0.494	-0.4	-0.603	-0.142	-0.717 *
**Fungi**	**Ascomycota**	-0.714 *	-0.683 *	0.226	-0.2	0.31	0.837 **	0.217
	**Basidiomycota**	0.681 *	0.65	-0.209	0.167	-0.326	-0.854 **	-0.117
	**Ciliophora**	0.328	0.4	0.268	-0.533	-0.05	-0.025	-0.417
	**Zygomycota**	0.714 *	0.033	-0.393	-0.667 *	-0.603	-0.301	-0.65

^a^Domination phylum in both bacterial and fungal community, *Proteobacteria* in class level.

^b^ALP = Alkaline phosphatase, TN = total nitrogen, TP = total phosphorus, AN = available nitrogen, AK = available potassium, SMC = soil moisture content.

^c^* = the two indexes were significantly correlated at *P* < 0.05,

** = the two indexes were significantly correlated at *P* < 0.01.

The soil microbial community *α*-diversity indexes have no correlation ship with soil type, soil enzyme activities and soil bacterial and fungal phylum (S3 and S4 Tables). However, both reads number and coverage has extremely negative correlation with TP (*r* = -0.883 and -0.917, *P <* 0.01, respectively) and positively correlation with TK (*r* = 0.720, *P <* 0.05 and *r* = 0.803, *P <* 0.01, respectively).

## Discussion

The objective of this study was to compare the differences of continuous monoculture of cover crops on soil microbial species composition and other related parameters of soil, and the differences in soil functions were better explained using both soil physicochemical test values and bacterial community structure data than using soil tests alone [18].

### Effects of different cover crops on soil physicochemical properties and soil enzymes activities

Soil physicochemical properties were influenced by many ways, such as fertilization [19], field management [12, 16], cover crops [31], and rotation system [32] and so on. In this study, after 5 years of monocropping, the soil nutrient content differed in soils, suggested that monocropping of different cover crops influenced soil nutrient contents, and TN, AN, AP and AK were significantly higher in MZ soil (Table 1). This was inconsistent with the research which compared monocropping with natural fallow treatment of ten-year fertilized winter wheat, and no significant difference in TN were observed [33]. The total nutrient differences of three soils were probably caused by the nutrient uptake efficiency and ratio of different types of cover crops [34]. The absorption ratio of N: P: K was about 2.2:1:2.9 in Gramineae (Proso millet), 1.6:1:1.9 in Legume (Common bean), and 2.5:1:3.2 in Polygonaceae (Common buckwheat) [2, 35, 36], this suggested that at the same soil fertility level (without fertilization), common bean consumed P more and common buckwheat consumed K more, and long-term monocropping without fertilization increases soil nutrient imbalance [19, 31]. Soil pH is mainly related to the species and root distribution of plants [37]. Legumes lower the soil pH when they fix N2 in the air [38]. Microorganisms can exhale CO_2_ through respiration and secrete some organic acids to cause the change of rhizosphere pH [23].

Soil enzyme play a pivotal role in soil biochemical processes, and had been used as indicators in the evaluation of soil recovery conditions in different ecosystem [14], moreover, they had been reported to be sensitive indicators of changes in soil quality [6]. In this research, the significant differences of soil enzyme activities (Fig 1A, 1B and 1C) indicated that monocropping of different cover crops influenced soil enzyme activities. These were consistent with V. Acosta-Martínez *et al*.’s research [6], in which they found increases in sensitive soil quality parameters under alternative management compared with cotton monoculture. Soil catalase activities indicate the ability to remove hydrogen peroxide toxicity and can also reflect the soil quality and the total metabolic activity of soil microorganisms from the side [39]. Soil urease is an obligate enzyme that hydrolyzes urea and produces ammonia and carbonic acid and soil urease activities reflect the conversion capacity of soil organic nitrogen to available nitrogen and the supply capacity of soil inorganic nitrogen [40]. Soil ALP can catalyze the hydrolysis of monophosphate and phosphodiester in soil to form inorganic phosphorus which can be absorbed by plants, and ALP activities can be used to characterize soil phosphate status [41]. The results (Fig 1A, 1B and 1C) indicated that monocropped YD soil had lower microorganism metabolic activities and soil quality and had higher capacity to supply AN and AP. However, the soil AN and AP content of YD were lower than that of MZ. These may cause by YD had a higher demand for AN and AP, although the capacities of supply were higher, the remaining content in soil were lower. As for soil sucrose activities (Fig 1D), no significant difference were obtained, which indicating that monocropping of different crops had no significant effect on soil soluble nutrient content. Moreover, root exudates can influence soil available nutrients content [42].

### Effects of different cover crops on soil microbial community composition

The microbial community plays a key role in soil aggregate stability, soil organic matter formation, and the potential for substrate metabolism from the degradation of plant residues, organic amendments, and xenobiotic [18, 43]. Study of different tillage system of soybean showed both fungal and bacterial community were influenced by the tillage system [5] and soil microbial community and functioning might respond to management and land use [12, 15]. Because most soil microbial taxa are rare [44], it is impossible to detect the full extent of bacterial diversity in a single soil, even if a full pyrosequencing was conducted [45, 46]. Therefore, the pyrosequencing depth of this study was suitable for the purpose of this research (S1 and S2 Figs).

It is now widely accepted that bacterial communities are composed by assembly resident taxa, those being slightly affected by environmental variables, and by occasional / fluctuating taxa, those varying between samples. In Adria L. Fernandez’s [18] research indicated that bacterial OTU richness was both positively and negatively associated with specific nutrient-cycling functions. The obtained OTUs found difference in the richness (α-diversity) indexes Ace and Chao1 as affected by the management and land use evaluated [47]. However, data analysis did not get any OTU number, Chao 1 and Ace differences between soil samples in our study, indicated that no significant differences of richness and diversity were obtained [47], and this was consistent with 10-years winter wheat soil in Loess Plateau [48]. Moreover, the QM and YD soils were much similar according to heatmap analysis (Fig 4).

The RA of bacterial and fungal phyla reported in this study generally agreed with previously reported pyrosequencing analysis results of soil microcommunities [21, 23, 49, 50]. Although the order of the bacterial communities is rare, the first five phyla were the same in different soils. The top five bacteria were *Proteobacteria*, *Acidobacteria*, *Chloroflexi*, *Gemnatimonadetes*, and *Bacteroidetes* (expect *bacterial-norank*), and this result was generally agreed with several results in different studies [21, 49, 50]. Whereas, the relative average abundance of *Chloroflexi* and *Gemmatimonadetes* in our samples was nearly 20 and 10%, which is 4 times and 2 times higher than those phylum reported in black soils (nearly 5%) [49], 20 times and 10 times higher than those phylum reported in America soils [50] and 29 soils reported by Chu *et al*. (less than 1%) [51], respectively. This might cause by different SMC and soil organic matter in different soils [20] and land use [52]. In addition, the RA of *Planctomycetes* (1%) is one fifth of that in black soil [49] and roughly equal of that in 88 soils across America [50]. This might cause by different soil ventilation [23] in different soils.

The four dominant fungal phylum was consistent with different fallow system in winter wheat [48], grass land [31] and black soil [53], however, the RA of *Ascomycota* in their studies was about 50–70%, much less than that in our result (about 80–90%). The diversity of fungal communities and distribution of several abundant fungal taxa were significantly related to the soil carbon content in black soils, and CCA plots indicated that fungal community composition was strongly affected by soil carbon content followed by soil pH [53]. This situation may cause by soil parameter differences [25] and land use (cover crop and management) [54]. In addition, *Glomeromycota* is one of the arbuscular mycorrhiza fungus [55], was significantly higher in YD soil, this situation consistent with the character of legume species.

RDA (*P <* 0.0001) indicated that SMC and pH were the most strongly predictive of community composition in both bulk and rhizosphere soil (*P <* 0.05) [18] and *Acidobacteria* showed strong negative relation with pH [22, 56]. However, in our research, RDA indicated that AN and AK were the most strongly predictive of community composition (Fig 5), and *Acidobacteria* had no relationship with pH (Fig 5A and S3 Table) which was consistent with study in agricultural black soil in China [53]. These may due to the SMC had no significant difference among soils and the pH of soils were between 8.7 and 8.8, with no big difference and no big influence on the community composition, therefore, nutrient factors (AN and AK) become the strongest predictive factors.

Overall, the differences may due to different cover crops, and studies suggested that there are many kinds of root exudates, including sugars, amino acids and vitamins, provide sufficient nutrition for the growth and reproduction of rhizosphere microorganisms, and also affect the species, quantity and distribution of soil microorganisms in rhizosphere [57, 58]. On the one hand, long-time monoculture enrich root exudates and provide an environment that is not suitable for the survival of beneficial microorganisms [59], on the other hand, the abundance of nutrient elements in soil directly affects the composition and quantity of root exudates [35, 36]. Moreover, long-time continuous monocropping results in the accumulation of the same root exudates, which providing a specific soil environment for the selection of soil microorganisms [12]. Therefore, the significantly increase of *Proteobacteria* indicated that monocropping of plant significantly decrease the soil quality, according to high RA of *Proteobacteria* in QM and YD soils, indicated that QM and YD are more sensitive to monocropping.

## Conclusions

In this study, monocropping of different cover crops (proso millet, common buckwheat and common bean) resulted in significantly difference in soil total and available nutrient content, soil pH and soil ALP, soil urease and catalase activities. In the meantime, monocropping of these three cover crops affected soil bacterial and fungal community compositions and distributions, but not differ the bacterial and fungal richness and diversity. Phylum *Proteobacteria*, *Acidobacteria*, *Chloroflexi* and *Gemnatimonadetes* were the most abundant bacterial phylum and *Ascomycota* was the domination fungal phyla in all three soils. Furthermore, RDA indicated that soil AN and AK were the strongest factors in both bacterial and fungal communities, soil nitrogen and water content might be the key factors in bacterial community formation, and soil phosphorus might be the key factor in fungal community formation. All the results suggested that monocropping of different cover crops influenced soil physicochemical properties and further influenced soil microbial composition. Moreover, continuous monocropping system was not recommended, and certainly not suggested in common bean and common buckwheat.

## Supporting information

S1 Fig The rarefaction curve of bacterial and fungal OTUs in three soils. MZ: continuous cropping proso millet for 5 years, YD: continuous cropping common bean for 5 years, QM: continuous cropping common buckwheat for 5 years. “01”, represent for bacteria, “02” represent for fungi, A, B and C represent different repetitions.
(TIF)

S2 Fig The Shannon-Wiener curve of bacterial and fungal OTUs in three soils. MZ: continuous cropping proso millet for 5 years, YD: continuous cropping common bean for 5 years, QM: continuous cropping common buckwheat for 5 years. “01”, represent for bacteria, “02” represent for fungi, A, B and C represent different repetitions.
(TIF)

S1 Table Significance the relative abundance differences of orders in Proteobacteria. Values are presented as the mean and standard deviation (n = 3). Significant differences were analyzed by one-way ANOVA Duncan’s test at *P* < 0.05 (SPSS 16.0). MZ, YD and QM represent soil samples collected from the fifth year continuous cropping of proso millet, common bean and common buckwheat history, respectively.
(PDF)

S2 Table Significant differences of bacterial and fungal phylum relative abundance. Values are presented as the mean ± standard deviation (n = 3). Means followed by the same letter are not significantly different as detected by Duncan’s test at *P* < 0.05 and Capital letters represent significant difference at *P* < 0.01. MZ, YD and QM represent soil samples collected from the fifth year continuous cropping of proso millet, common bean and common buckwheat history, respectively.
(PDF)

S3 Table Spearman’s rank correlations (r) between bacteria abundant taxa (RA >1% in all samples combined) and soil properties. Correlation ship was analyzed by spearman (SPSS 16.0). ALP alkaline phosphatase; TN total nitrogen; TP total phosphorus; TK total potassium; AN available nitrogen; AK available potassium; AP available phosphorus; SMC soil moisture content. *represent for significant difference at *P* < 0.05 level; ** represent for significant difference at *P* < 0.01 level. Phylum with 8 initials.
(PDF)

S4 Table Spearman’s rank correlations (r) between fungi abundant taxa (RA >1% in all samples combined) and soil properties. Correlation ship was analyzed by spearman (SPSS 16.0). ALP alkaline phosphatase; TN total nitrogen; TP total phosphorus; TK total potassium; AN available nitrogen; AK available potassium; AP available phosphorus; SMC soil moisture content. *represent for significant difference at *P* < 0.05 level; ** represent for significant difference at *P* < 0.01 level.
(PDF)

## Abbreviations

AK: available potassium
ALP: alkaline phosphatase
AN: available nitrogen
AP: available phosphorus
MZ: proso millet
QM: common buckwheat
SMC: soil moisture content
TK: total potassium
TN: total nitrogen
TP: total phosphorus
YD: common bean
