# Supplementary figures and images for: Soil properties, bacterial and fungal community compositions and the key factors after 5-year continuous monocropping of three minor crops
Source: PLoS One. 2020 Aug 24;15(8):e0237164. doi: 10.1371/journal.pone.0237164 (PMC7446844; doi:10.1371/journal.pone.0237164)

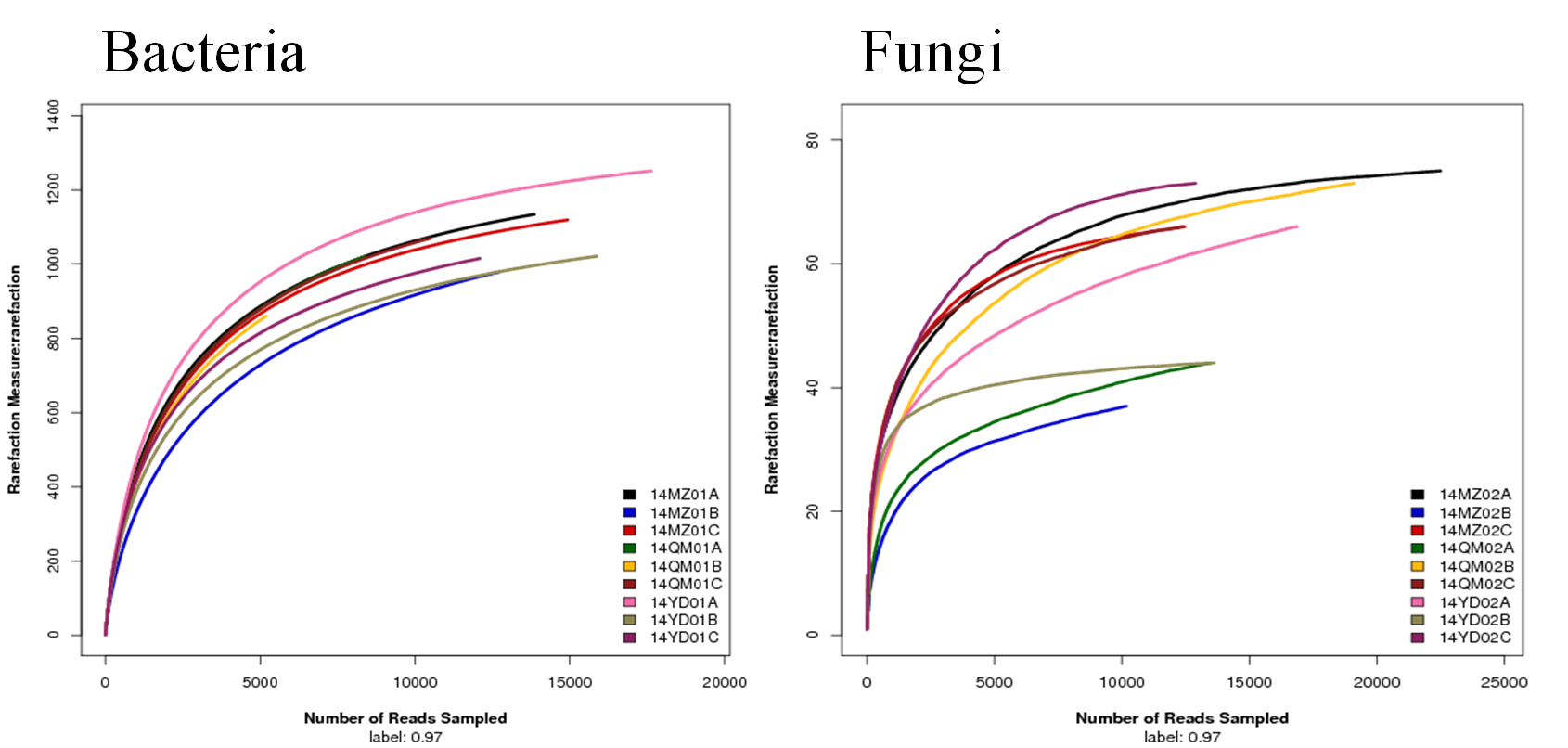

Supplement: S1 Fig — MZ: continuous cropping proso millet for 5 years, YD: continuous cropping common bean for 5 years, QM: continuous cropping common buckwheat for 5 years. “01”, represent for bacteria, “02” represent for fungi, A, B and C represent different repetitions. (TIF) [file pone.0237164.s001.tif]

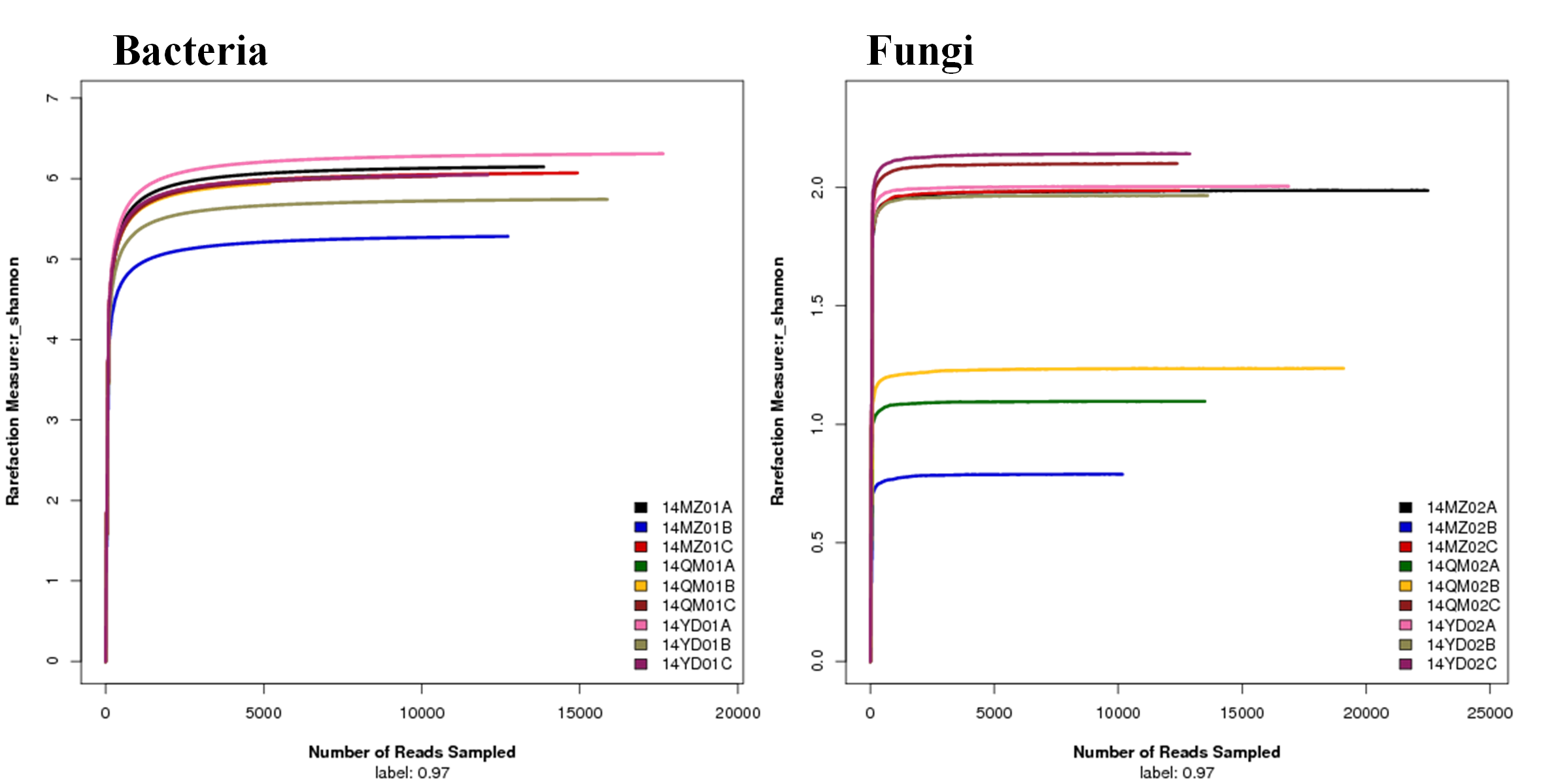

Supplement: S2 Fig — MZ: continuous cropping proso millet for 5 years, YD: continuous cropping common bean for 5 years, QM: continuous cropping common buckwheat for 5 years. “01”, represent for bacteria, “02” represent for fungi, A, B and C represent different repetitions. (TIF) [file pone.0237164.s002.tif]
