# Supplementary material for: Soil properties, bacterial and fungal community compositions and the key factors after 5-year continuous monocropping of three minor crops
Source: PLoS One. 2020 Aug 24;15(8):e0237164. doi: 10.1371/journal.pone.0237164 (PMC7446844; doi:10.1371/journal.pone.0237164)
Supplement: S1 Table — Values are presented as the mean and standard deviation (n = 3). Significant differences were analyzed by one-way ANOVA Duncan’s test at P < 0.05 (SPSS 16.0). MZ, YD and QM represent soil samples collected from the fifth year continuous cropping of proso millet, common bean and common buckwheat history, respectively. (PDF) [file pone.0237164.s003.pdf]

	Taxon	MZ	SD of MZ [a]	Significance	QM	SD of QM	Significance	YD	SD of YD	Significance
**Bacteria**	**Acidobacteria**	24.20%	16.31%		14.58%	3.31%		11.60%	7.65%	
	**Proteobacteria**	23.74%	11.37%		27.03%	8.48%		28.59%	5.24%	
	**Chloroflexi**	20.42%	6.23%		17.18%	9.50%		18.45%	6.70%	
	**Bacteria_norank**	13.67%	7.72%		17.61%	5.37%		15.29%	3.56%	
	**Gemmatimonadetes**	6.67%	3.11%		10.15%	4.14%		11.33%	3.89%	
	**Bacteroidetes**	2.87%	1.24%		2.95%	1.12%		2.03%	0.89%	
	**Firmicutes**	2.04%	0.84%		2.01%	1.26%		5.25%	6.56%	
	**Planctomycetes**	1.68%	1.09%		1.31%	0.55%		1.43%	0.37%	
	**Nitrospirae**	1.66%	0.39%		2.72%	0.96%		3.54%	1.21%	
	**Cyanobacteria**	0.94%	0.52%		2.09%	3.10%		0.48%	0.74%	
	**Candidate_division_TM7**	0.65%	0.23%		0.48%	0.26%		0.42%	0.25%	
	**Candidate_division_WS3**	0.58%	0.43%		0.75%	0.65%		0.64%	0.32%	
	**Verrucomicrobia**	0.35%	0.27%		0.18%	0.15%		0.13%	0.18%	
	**JL-ETNP-Z39**	0.21%	0.16%		0.08%	0.03%		0.13%	0.13%	
	**Bacteria_unclassified**	0.10%	0.04%	* b	0.26%	0.04%	* a	0.24%	0.10%	* ab
	**Chlorobi**	0.10%	0.08%		0.09%	0.07%		0.06%	0.04%	
	**WCHB1-60**	0.06%	0.08%		0.08%	0.09%		0.02%	0.00%	
	**TM6**	0.05%	0.02%		0.07%	0.03%		0.04%	0.03%	
	**Armatimonadetes**	0.04%	0.03%		0.06%	0.01%		0.07%	0.02%	
	**Caldiserica**	0.04%	0.02%	* b	0.14%	0.04%	* a	0.10%	0.07%	* ab
	**Deinococcus-Thermus**	0.03%	0.00%		0.03%	0.02%		0.03%	0.02%	
	**Candidate_division_OD1**	0.03%	0.02%		0.07%	0.05%		0.05%	0.04%	
	**Thermotogae**	0.02%	0.01%		0.03%	0.02%		0.02%	0.01%	
	**Candidate_division_BRC1**	0.02%	0.02%		-	-		0.01%	0.01%	
	**Elusimicrobia**	0.02%	0.02%		0.00%	0.01%		0.01%	0.01%	

	**SM2F11**	0.02%	0.02%		0.02%	0.02%		0.03%	0.02%	
	**TA06**	0.01%	0.01%		0.00%	0.01%		0.01%	0.01%	
**Fungi**	**Ascomycota**	91.665%	6.280%		77.089%	13.071%		80.588%	3.993%	
	**Basidiomycota**	3.434%	2.618%		18.278%	13.750%		12.152%	5.494%	
	**Ciliophora**	1.924%	1.737%		0.888%	0.515%		1.534%	0.630%	
	**Zygomycota**	1.368%	1.283%		1.379%	0.944%		3.523%	1.106%	
	**Fungi_unclassified**	0.912%	0.447%		0.887%	0.528%		0.814%	0.237%	
	**Eukaryota_unclassified**	0.149%	0.141%		0.129%	0.107%		0.175%	0.140%	
	**Glomeromycota**	0.134%	0.111%	* b	0.067%	0.058%	* b	0.466%	0.187%	* a
	**Chytridiomycota**	0.134%	0.123%		0.071%	0.060%		0.045%	0.021%	
	**Fungi_incertae_sedis**	0.060%	0.036%	* b	1.014%	0.418%	* a	0.127%	0.072%	* b
	**Centrohelida_norank**	0.041%	0.038%		0.015%	0.007%		0.005%	0.009%	
	**Cercozoa**	0.036%	0.034%		0.016%	0.016%		0.007%	0.006%	
	**Fungi_uncultured**	0.024%	0.035%		0.010%	0.013%		0.004%	0.007%	
	**Blastocladiomycota**	0.023%	0.020%		0.032%	0.017%		0.014%	0.016%	
	**Schizoplasmodiida**	0.021%	0.020%		0.007%	0.012%		0.013%	0.022%	
	**Bicosoecida**	0.016%	0.028%		0.007%	0.012%		0.008%	0.013%	
	**Amoebozoa_unclassified**	0.015%	0.022%		-	-		0.004%	0.007%	
	**Choanomonada**	0.012%	0.014%		0.017%	0.009%		0.182%	0.174%	
	**WIM5**	0.010%	0.013%		-	-		0.004%	0.007%	
	**Tubulinea**	0.009%	0.015%		0.002%	0.004%		0.002%	0.003%	
	**Nucleariida**	0.009%	0.009%		0.009%	0.008%		0.047%	0.051%	
	**Phragmoplastophyta**	0.006%	0.005%	* b	0.023%	0.020%	* b	0.187%	0.055%	* a
	**Acanthocystidae**	0.001%	0.003%		0.002%	0.003%		0.005%	0.009%	
	**Fungi_norank**	-	-		0.027%	0.047%		-	-	
	**Heterolobosea**	-	-		-	-		0.018%	0.031%	

	**Metazoa**	-	-	0.034%	0.055%	-	-
	**Ochrophyta**	-	-	-	-	0.076%	0.132%

Values are presented as the mean and standard deviation (n = 3). Significant differences were analyzed by one-way ANOVA Duncan's test at $P < 0.05$ (SPSS 16.0). MZ, YD and QM represent soil samples collected from the fifth year continuous cropping of proso millet, common bean and common buckwheat history, respectively.
