# Supplementary material for: Soil properties, bacterial and fungal community compositions and the key factors after 5-year continuous monocropping of three minor crops
Source: PLoS One. 2020 Aug 24;15(8):e0237164. doi: 10.1371/journal.pone.0237164 (PMC7446844; doi:10.1371/journal.pone.0237164)
Supplement: S2 Table — Values are presented as the mean ± standard deviation (n = 3). Means followed by the same letter are not significantly different as detected by Duncan’s test at P < 0.05 and Capital letters represent significant difference at P < 0.01. MZ, YD and QM represent soil samples collected from the fifth year continuous cropping of proso millet, common bean and common buckwheat history, respectively. (PDF) [file pone.0237164.s004.pdf]

		α-Proteobacteria	β-Proteobacteria	δ-Proteobacteria	γ-Proteobacteria
**In Proteobacteria**	**MZ**	57.56%±2.80% A	16.82%±1.93% b	14.57%±2.45% b	11.04%±1.16% a
	**QM**	29.92%±2.76% B	34.00%±8.69% a	24.50%±6.08% a	11.56%±5.77% a
	**YD**	35.36%±5.55% B	24.75%±6.53% ab	22.72%±0.81% a	17.14%±6.49% a
**In Total**	**MZ**	13.81%±6.93% a	3.93%±1.85% a	3.36%±1.59% b	2.57%±1.21% a
	**QM**	10.37%±5.71% a	12.97%±4.99% a	8.00%±6.36% a	5.12%±3.18% a
	**YD**	9.93%±0.68% a	7.23%±3.03% a	6.47%±0.99% ab	4.95%±2.21% a

Values are presented as the mean ± standard deviation (n = 3). Means followed by the same letter are not significantly different as detected by Duncan's test at $P < 0.05$ and Capital letters represent significant difference at $P < 0.01$. MZ, YD and QM represent soil samples collected from the fifth year continuous cropping of proso millet, common bean and common buckwheat history, respectively.
