# Supplementary material for: Soil properties, bacterial and fungal community compositions and the key factors after 5-year continuous monocropping of three minor crops
Source: PLoS One. 2020 Aug 24;15(8):e0237164. doi: 10.1371/journal.pone.0237164 (PMC7446844; doi:10.1371/journal.pone.0237164)
Supplement: S3 Table — Correlation ship was analyzed by spearman (SPSS 16.0). ALP alkaline phosphatase; TN total nitrogen; TP total phosphorus; TK total potassium; AN available nitrogen; AK available potassium; AP available phosphorus; SMC soil moisture content. *represent for significant difference at P < 0.05 level; ** represent for significant difference at P < 0.01 level. Phylum with 8 initials. (PDF) [file pone.0237164.s005.pdf]

	Soil type	Acidobac	Bacteria_no	Bacteroi	Chlorofl	Firmicut	Gemmati	Nitrospi	Planctom	Proteoba	α-Proteo	β-Proteo	δ-Proteo	γ-Proteo	ALP	Catalase	Urease	Sucrose	TN	TP	TK	AN	AP	AK	pH	SMC	Reads	OTUs	Ace	Chao	Coverage	Shannon
**Acidobac**	-0.422	1																														
**Bacteria_no**	-0.053	-0.567	1																													
**Bacteroi**	-0.369	-0.017	0.617	1																												
**Chlorofl**	-0.158	0.667*	-0.417	-0.417	1																											
**Firmicut**	0.211	-0.617	0.083	0.15	-0.783*	1																										
**Gemmati**	0.685*	-0.683*	-0.05	-0.333	-0.45	0.633	1																									
**Nitrospi**	0.791*	-0.667*	0.017	-0.3	-0.417	0.483	0.933**	1																								
**Planctom**	0.053	0.6	-0.383	-0.533	.917**	-0.850**	-0.4	-0.333	1																							
**Proteoba**	0.211	-0.883**	0.533	0.3	-0.717*	0.733*	0.6	0.5	-0.767*	1																						
**α-Proteo**	-0.158	-0.567	0.467	0.45	-0.45	0.667*	0.183	0.083	-0.683*	0.767*	1																					
**β-Proteo**	0.474	-0.733*	0.533	0.3	-0.717*	0.367	0.567	0.683*	-0.583	0.683*	0.2	1																				
**δ-Proteo**	0.685*	-0.700*	0.117	-0.2	-0.417	0.483	0.933**	0.867**	-0.333	.0667*	0.15	0.683*	1																			

**γ-Proteo**	0.474	-0.733*	-0.05	-0.367	-0.433	0.717*	0.933**	0.783*	-0.45	0.700*	0.383	0.417	0.850**	1													
**ALP**	0.850**	-0.336	0.168	-0.176	-0.034	0.16	0.412	0.496	0.126	0.143	0.034	0.218	0.412	0.252	1												
**Catalase**	-0.738*	0.15	0.5	0.5	-0.133	-0.183	-0.583	-0.617	-0.15	-0.133	0.05	-0.15	-0.55	-0.5	-0.496	1											
**Urease**	0.632	-0.25	-0.05	-0.4	0.017	0.05	0.133	0.317	0.183	-0.05	-0.017	0.067	0.017	0.067	0.689*	-0.467	1										
**Sucrose**	-0.158	0.033	0.633	0.5	-0.033	-0.117	-0.3	-0.283	-0.017	-0.083	0.1	-0.067	-0.25	-0.367	0.261	0.65	-0.133	1									
**TN**	-0.767*	0.377	0.167	0.251	0.494	-0.393	-0.661	-0.653	0.218	-0.293	0.243	-0.561	-0.686*	-0.494	-0.46	0.544	-0.301	0.343	1								
**TP**	-0.369	0.183	0.35	0.267	0.067	-0.533	-0.35	-0.367	0.2	-0.167	-0.433	0.133	-0.1	-0.4	-0.412	0.6	-0.6	0.317	0.1	1							
**TK**	0.318	-0.117	-0.017	-0.126	0.31	0.084	0.075	0.176	0.142	0.075	0.435	-0.184	-0.025	0.109	0.536	-0.494	0.603	-0.025	0.248	-0.770*	1						
**AN**	-0.873**	0.51	0.059	0.151	0.552	-0.561	-0.795*	-0.837**	0.368	-0.418	0	-0.636	-0.753*	-0.603	-0.637	0.628	-0.418	0.226	0.903**	0.377	-0.067	1					
**AP**	-0.482	0.136	-0.034	0.119	0.288	0.051	-0.407	-0.424	-0.017	0	0.593	-0.509	-0.525	-0.17	-0.214	0.068	0.119	-0.051	0.732*	-0.509	0.647	0.553	1				
**AK**	-.714*	0.209	-0.243	0.126	-0.084	0.218	-0.427	-0.586	-0.251	0.025	0.36	-0.435	-0.519	-0.142	-0.667*	0.285	-0.226	-0.318	0.37	-0.142	-0.109	0.492	0.604	1			
**pH**	0.266	-0.034	-0.084	-0.092	-0.345	-0.008	0.277	0.235	-0.05	-0.025	-0.588	0.403	0.387	0.109	-0.038	0.034	-0.244	-0.084	-0.709*	0.563	-0.823**	-0.435	-0.940**	-0.3	1		
**SMC**	-0.369	0.367	0.133	0.633	-0.067	-0.217	-.683*	-0.517	-0.117	-0.183	0.083	0	-0.617	-0.717*	-0.286	0.25	0.033	0.017	0.259	0.067	0.025	0.276	0.322	0.385	-0.202	1	
**Reads**	0.158	0.2	-0.45	-0.25	0.2	0.283	0	0	0.067	-0.217	0.233	-0.517	-0.267	0.05	0.361	-0.367	0.533	-0.067	0.126	-0.883**	0.720*	-0.1	0.576	0.209	-0.605	0.017	1

**OTUs**	0	0.283	-0.05	0.15	0.217	0.15	-0.117	-0.167	0.067	-0.233	0.217	-0.5	-0.267	-0.133	0.403	0.067	0.1	0.583	0.318	-0.433	0.494	0.067	0.356	-0.092	-0.471	-0.083	0.700*	1				
**Ace**	-0.264	0.6	-0.017	0.3	0.517	-0.3	-0.467	-0.533	0.367	-0.433	-0.017	-0.617	-0.433	-0.483	0.143	0.233	-0.25	0.617	0.494	0.05	0.243	0.41	0.271	-0.067	-0.336	0.1	0.317	0.800**	1			
**Chao**	-0.264	0.6	-0.017	0.3	0.517	-0.3	-0.467	-0.533	0.367	-0.433	-0.017	-0.617	-0.433	-0.483	0.143	0.233	-0.25	0.617	0.494	0.05	0.243	0.41	0.271	-0.067	-0.336	0.1	0.317	0.800**	1.000**	1		
**Coverage**	0.264	0.067	-0.333	-0.217	0.15	0.333	0.1	0.117	0.017	-0.1	0.317	-0.4	-0.15	0.133	0.479	-0.417	0.583	-0.017	0.092	-0.917**	0.803**	-0.184	0.559	0.092	-0.639	-0.033	0.983**	0.717*	0.3	0.3	1	
**Shannon**	0.053	0.05	0.326	0.494	0.126	0.126	-0.1	-0.126	-0.059	0.134	0.485	-0.159	-0.059	-0.117	0.46	-0.008	0	0.586	0.311	-0.276	0.58	0.042	0.374	-0.189	-0.532	0.109	0.393	0.787*	0.770*	0.770*	0.477	1
**Simpson**	-0.158	0.15	-0.35	-0.5	0.033	-0.283	-0.067	0	0.2	-0.4	-0.617	-0.033	-0.167	-0.083	-0.471	0.183	0	-0.367	-0.109	0.3	-0.552	0.117	-0.322	0.126	0.445	-0.1	-0.283	-0.567	-0.567	-0.567	-0.383	-0.929**

Correlation ship was analyzed by spearman (SPSS 16.0). ALP alkaline phosphatase; TN total nitrogen; TP total phosphorus; TK total potassium; AN available nitrogen; AK available potassium; AP available phosphorus; SMC soil moisture content.

*represent for significant difference at *p*<0.05 level; ** represent for significant difference at *p*<0.01 level. Phylum with 8 initials.
