# Supplementary material for: Soil properties, bacterial and fungal community compositions and the key factors after 5-year continuous monocropping of three minor crops
Source: PLoS One. 2020 Aug 24;15(8):e0237164. doi: 10.1371/journal.pone.0237164 (PMC7446844; doi:10.1371/journal.pone.0237164)
Supplement: S4 Table — Correlation ship was analyzed by spearman (SPSS 16.0). ALP alkaline phosphatase; TN total nitrogen; TP total phosphorus; TK total potassium; AN available nitrogen; AK available potassium; AP available phosphorus; SMC soil moisture content. *represent for significant difference at P < 0.05 level; ** represent for significant difference at P < 0.01 level. (PDF) [file pone.0237164.s006.pdf]

	Soil type	Ascomycota	Basidiomycota	Ciliophora	Fungi_unclassified	Zygomycota	ALP	Catalase	Urease	Sucrose	TN	TP	TK	AN	AP	AK	pH	SMC	Reads	OTUs	Ace	Chao	Coverage	Shannon
**Ascomycota**	-0.527	1																						
**Basidiomycota**	0.527	-0.983**	1																					
**Ciliophora**	0.053	-0.133	0.1	1																				
**Fungi_unclassified**	-0.105	-0.05	0.017	0.517	1																			
**Zygomycota**	0.632	-0.267	0.2	0.65	0.317	1																		
**ALP**	0.850**	-0.714*	0.681*	0.328	0	0.714*	1																	
**Catalase**	-0.738*	-0.067	0.05	0.1	0.017	-0.533	-0.496	1																
**Urease**	0.632	-0.3	0.317	0.217	-0.45	0.4	0.689*	-0.467	1															
**Sucrose**	-0.158	-.683*	0.65	0.4	0.267	0.033	0.261	0.65	-0.133	1														
**TN**	-0.767*	0.226	-0.209	0.268	0.402	-0.393	-0.46	0.544	-0.301	0.343	1													
**TP**	-0.369	-0.2	0.167	-0.533	0	-0.667*	-0.412	0.6	-0.6	0.317	0.1	1												
**TK**	0.318	-0.059	0.084	0.51	0.243	0.485	0.536	-0.494	0.603	-0.025	0.248	-0.770*	1											
**AN**	-0.873**	0.31	-0.326	-0.05	0.192	-0.603	-0.637	0.628	-0.418	0.226	0.903**	0.377	-0.067	1										
**AP**	-0.482	0.509	-0.492	0.424	0.186	-0.017	-0.214	0.068	0.119	-0.051	0.732*	-0.509	0.647	0.553	1									
**AK**	-0.714*	0.837**	-0.854**	-0.025	-0.259	-0.301	-0.667*	0.285	-0.226	-0.318	0.37	-0.142	-0.109	0.492	0.604	1								
**pH**	0.266	-0.252	0.227	-0.555	-0.345	-0.134	-0.038	0.034	-0.244	-0.084	-.709*	0.563	-0.823**	-0.435	-0.940**	-0.3	1							
**SMC**	-0.369	0.217	-0.117	-0.417	-0.55	-0.65	-0.286	0.25	0.033	0.017	0.259	0.067	0.025	0.276	0.322	0.385	-0.202	1						
**Reads**	0.158	0.067	-0.05	0.5	-0.05	0.567	0.361	-0.367	0.533	-0.067	0.126	-0.883**	.720*	-0.1	0.576	0.209	-0.605	0.017	1					
**OTUs**	0	-0.367	0.35	0.6	0.333	0.517	0.403	0.067	0.1	0.583	0.318	-0.433	0.494	0.067	0.356	-0.092	-0.471	-0.083	0.700*	1				
**Ace**	-0.264	-0.333	0.3	0.15	0.35	0.083	0.143	0.233	-0.25	0.617	0.494	0.05	0.243	0.41	0.271	-0.067	-0.336	0.1	0.317	0.800**	1			
**Chao**	-0.264	-0.333	0.3	0.15	0.35	0.083	0.143	0.233	-0.25	0.617	0.494	0.05	0.243	0.41	0.271	-0.067	-0.336	0.1	0.317	0.800**	1.000**	1		
**Coverage**	0.264	-0.017	0.033	0.583	0.033	0.65	0.479	-0.417	0.583	-0.017	0.092	-0.917**	0.803**	-0.184	0.559	0.092	-0.639	-0.033	0.983**	0.717*	0.3	0.433	1	
**Channon**	0.053	-0.343	0.326	0.452	0.452	0.377	0.46	-0.008	0	0.586	0.311	-0.276	0.58	0.042	0.374	-0.189	-0.532	0.109	0.393	0.787*	0.770*	0.015	0.194	1
**Simpson**	-0.158	0.183	-0.15	-0.35	-0.4	-0.417	-0.471	0.183	0	-0.367	-0.109	0.3	-0.552	0.117	-0.322	0.126	0.445	-0.1	-0.283	-0.567	-0.567	0.112	0.308	0

Correlation ship was analyzed by spearman (SPSS 16.0). ALP alkaline phosphatase; TN total nitrogen; TP total phosphorus; TK total potassium; AN available nitrogen; AK available potassium; AP available phosphorus; SMC soil moisture content.

*represent for significant difference at p<0.05 level; ** represent for significant difference at p<0.01 level.
